# Identification of recycling potential of construction and demolition waste: challenges and opportunities in the Greater Dhaka area

**DOI:** 10.1007/s10661-025-14081-8

**Published:** 2025-05-10

**Authors:** Shama E. Haque, Nazmun Nahar, Nafisa N. Chowdhury, Lameesa Gazi-Khan, Tasriba K. Sayanno, Md. Abdul Muktadir, Md. Sazzadul Haque

**Affiliations:** 1 Department of Civil and Environmental Engineering, North South University, Dhaka, 1229 Bangladesh; 2 C3ER, BRAC University, Dhaka, 1212 Bangladesh; 3 Department of Civil and Environmental Engineering, Virginia Polytechnic Institute and State University, Manassas, VA 20110 USA

**Keywords:** Greater Dhaka, Construction and demolition waste, Recycling, Sustainability, Environmental impact

## Abstract

In the past five decades, Dhaka city, the capital of Bangladesh experienced urbanization in an unprecedented manner. The city has grown spatially in all directions to accommodate the urban population that resulted in accelerated growth of the construction and demolished floor areas in Dhaka and its surroundings, commonly known as the Greater Dhaka. As a result, the quantities of construction and demolition waste have increased significantly within this region. Through an onsite field investigation of 21 construction and 12 demolition project sites in Greater Dhaka, the study identified the waste generation rate to be approximately 463.67 kg and 90.31 kg per m^2^ floor area of demolition and construction projects, respectively. Projection based on this waste generation rate; the annual waste amount has been calculated for the eight districts of the Greater Dhaka region. The recycling potential identified through this study also estimated the economic benefits of the studied construction and demolition waste (CDW) materials for these eight districts for years 2022 to 2030. The findings of the present study are expected to assist the relevant stakeholders and policymakers to devise business development and legislative measures for the successful implementation of the sustainable waste management plan for the construction industry.

## Introduction

Globally, the rate of urbanization is increasing. In 2018, approximately 55.30% of people were reported to live in urban settlements which is projected to be 60% by 2030 (UN, 2018). Urbanization involves building activities that makes generation of Construction and Demolition Waste (CDW) an integral part of the urbanization process (Papamichael et al., 2023). Generally, CDW is defined to be the undesirable, heterogeneous residual matters that are particularly generated from different construction, renovation, and demolition activities of various infrastructures (Pallewatta et al., 2023). Different types of non-biodegradable items – concrete, mortar, metals, brick, wood, plastic, glass, tiles, excavated soils, asbestos, etc. – are the main constituents of CDW (Bianchini et al., 2020).

During the last five decades, Dhaka city, the capital of Bangladesh, has expanded across all directions and has transformed into a megacity. It is expected to be the home of approximately 28 million people by 2030, making it the fourth most populous megacity in the world (Ahmed & Montu, 2021). Dhaka, Bangladesh's economic center, is rapidly becoming more urbanized to meet the demands of its expanding population and the growing need for construction projects, utilities, and other services. Drainage, water supply, solid waste management, sewerage, sanitation, housing, and transportation; all these sectors result in the production of massive amounts of construction and demolition waste (CDW). Materials produced during new construction are classified as construction waste and materials generated from the tear-down of structures and buildings are referred to as demolition waste.

Previous studies on Dhaka's urban growth identified the city's difficulty in expanding horizontally due to flood risk or ongoing encroachment on natural wetlands and depressions (Ahmed & Bramley, 2015; Dewan & Yamaguchi 2009; Roy, 2009). As a result, in the city center, multi-story structures are the primary means of absorbing its 20 million residents, with 400,000 arriving each year (WB, 2007). Currently, Dhaka's densely populated metropolitan center is undergoing considerable reconstruction, with traditional six-story buildings being replaced with higher and heavier ones. Additional stories are being added to existing structures, frequently exceeding the allowable bearing strain on the subsoil supported by the foundations (Günther et al., 2015). Based on an analysis of Landsat-derived wetland maps, in recent years, the city has expanded spatially mostly by landfilling (76.67%) and encroachment (18.72%) that resulted in significant reduction of wetlands, rivers and *khals* (i.e., canals) (Habiba et al., 2011). Existing urban buildings underwent significant modifications to meet the growing demand for housing, office space, and other commercial setups. In general, upazilas (sub-district) within the Dhaka District, Narayanganj city, Bandar, and Rupganj Upazilas in Narayanganj District, Gazipur Sadar and Kaliakair Upazilas in Gazipur District, and Narsingdi Sadar in Narsingdi District are collectively referred as the Greater Dhaka area. The World Bank estimates that Greater Dhaka will have a population of 25.0 million people by 2035 and a per capita income of US$ 8,000 calculated based on the rate of 2015 (Bird et al., 2018).

A review of the existing literature on CDW indicates that there is a research gap in the waste generation rates (WGR) of Greater Dhaka area. Most of the research and technical resources related to the CDW management scenario in Dhaka City are informal (i.e., unpublished field survey reports, anecdotal evidence) and inadequate. For instance, using WGR and regression analysis, Islam et al. (2019) offered a method to estimate CDW generation in Dhaka city. According to the findings, WGR was 1615 kg/m^2^ for demolition activities and 63.74 kg/m^2^ for construction activities. In the fiscal year 2016, Dhaka city produced 1.28 million tons of waste (0.15 MT construction and 1.13 MT demolition), with concrete accounting for 60% of the debris, brick/block accounting for 21%, and mortar making up 9% (Islam et al., 2019). Haque et al. (2024) assessed the recycling potential and waste generation rates for the CDW in Dhaka and found that the average WGR for construction and demolition per unit area (m^2^) was approximately 73.90 kg and 575.0 kg, respectively. To the best of the authors’ knowledge, no study has been conducted to this date to investigate the WGR and recycling potential of the CDW and its adverse environmental impacts in the Greater Dhaka area.

Rapid urbanization and infrastructure development have made CDW a global issue due to its negative effects on sustainability's socioeconomic and environmental aspects (Daoud et al., 2021, 2023b). Developing countries (e.g., in Asia and Africa) still lack structured policy measures, recycling infrastructures, and economic incentives, despite the proven major challenges – environmental degradation, resource depletion, and GHG emissions—associated with the poor management of CDW (Daoud et al., 2023a; Ismail et al., 2023; Papamichael et al., 2023; Umar, 2022). In the regional context, Bangladesh faces similar challenges, particularly in the Greater Dhaka area, where rapid urban expansion and unprecedented rise in construction activities (Bird et al., 2018) have exacerbated an urgent need for effective CDW management infrastructure.

The objective of this present study is to address this gap by examining the current waste generation scenario in the Greater Dhaka region to assess the current waste disposal practices followed by the opportunities for efficient management. The study also investigates the recycling potential of the key waste components, evaluates the feasibility of recycling, and explores the economic benefits and challenges related to sustainable CDW management. Furthermore, the study aims to explore appropriate policy measures suitable to the region’s socioeconomic and infrastructural landscape for an improved and circular CDW management situation.

## Literature review

Globally, CDW management receives increasing attention from researchers as more and more people move to urban areas. For instance, Lu et al. (2011) conducted a study to investigate waste generation rate through on-site waste sorting and weighing in active construction projects in Shenzhen City in China. They found that the WGR ranged from 3.28 to 8.79 kg/m^2^ where concrete and timber are the major components of the generated waste. A study based in Bolivia examined CDW management, revealing that CDW generation rates in concrete residential buildings range from 91.90—113.30 kg/m2 during construction and 867.20—1064.80 kg/m2 during demolition (Ferronato et al., 2023). The findings suggest that La Paz city in Bolivia generates around 350,000 tons of CDW annually, recycling up to 71.10% of inert aggregates is feasible, with estimated costs ranging from 7.80 to 31.10 USD per ton of CDW, making an economically viable system possible, especially through source separation and hybrid stationary and mobile recycling facilities. The Chennai City in India generated 1.14 MT of CDW in 2013 (Ram & Kalidindi, 2017) whereas in Malaysia and Vietnam the reported amount was around 8.0 MT in 2021 and 5.30 MT in 2018, respectively (Menegaki & Damigos, 2018; Rahim et al., 2017). In Egypt, more than 40% of the materials’ costs in construction projects are found to be from CDW whereas these items are reported to be primarily disposed of indiscriminately (Daoud et al., 2023b).

Yuan and Shen (2011) investigated the latest research trend in the discipline by analyzing the publications from major international journals (2000–2009) and found that on-site surveys and local or regional case studies are the primary methods for CDW data collection and further investigation. UNEP (2015) reported that even though CDW waste is a global contributor to solid waste generation, it receives considerably less attention in the literature compared to municipal solid waste (MSW), which can be explained by the benign nature of CDW compared to that of MSW. Menegaki and Damigos (2018) revealed countries in the European Union (EU) generate a higher amount of CDW annually than any other Asian countries. Additionally, the findings indicate that the management of CDW has evolved into a social, environmental, and economic problem that needs urgent research for its subsequent valorization process. Table 1 presents a summary of some previously published research as an effort in developing current advancement on CDW management issues in different regions of the world.

**Table 1:** Summary of selected research on construction and demolition waste management

Source	Main objective	Summary of findings
Haque et al. (2023)	This paper focused on CDW management practices in Mymensingh, Bangladesh, and their effect on the neighboring environment	The study summarizes that among the 300 construction sites observed, 31% of the materials are recycled, 26% are reused, and the other items are classified as construction waste. The CDW is responsible for detrimental environmental effects such as bank scourging, and river flood levels
Moschen-Schimek et al. (2023)	A review of the waste recovery rates of CDW in the EU	According to the paper, CDW streams have a high potential for recycling. For these reasons, the European Commission established a CDW recovery target that member states must meet to submit their national CDW data to the EU
Tihomirovs et al. (2023)	A review of possible ways of utilizing waste glass in some useful products in the construction industry	The review highlights substantial efforts at exploring the potential use of waste glass as a substitute for natural aggregates and cement in various concrete applications. It also specifies that there are at least seven areas where demolition waste glass can be used such as concrete products, gypsum–cement composites, asphalt or concrete pavement etc
Datta et al. (2022)	Identification of construction waste-generating factors and management strategies for the construction industry of Bangladesh	The study involved a comprehensive examination, incorporating multiple surveys with diverse construction professionals. The survey utilized the Likert scale and prioritized waste-generating factors. The factor identified revealed that'deposited material in a public place'is the most significant contributor to waste on construction sites in Bangladesh
Zhang et al. (2022)	This paper explores the evolution of the waste hierarchy in Europe and how it compares with the circular economy	The study employed the waste hierarchy to investigate the management of CDW in Europe. The study highlighted the parallel evolution of the circular economy, emphasizing a novel approach to waste management by reevaluating, redesigning, and repurposing products to enhance resource efficiency and diminish waste generation throughout their life cycle
Ngieng et al. (2021)	Governmental policies in Malaysia, Singapore and Thailand, highlighting that concrete production contributes to roughly 10% of the total synthetic CO_2_ emission	The paper summarizes that governments in Southeast Asia have implemented various measures, including legislative acts, rules and incentives to mitigate the adverse effects of construction on the environment
Makul et al. (2021)	This review article discussed the lack of practical application of recycled concrete in Southeast Asia	The paper highlights while this region’s governments have initiated rules to foster sustainable concrete, there's a lack of awareness and economic feasibility that hinders the practical implementation of recycled aggregate concrete (RAC)
Prema (2021)	The effectiveness of existing legislation of India, in regulating the construction sector and how far they comply with the international standard of protecting the environment	The findings of the study indicate that CDW waste presents a significant environmental threat in India. Despite legislative reforms in the country, comprehensive CDW waste management remains limited, demanding a broader, more adaptable approach to tackle India's CDW waste challenge
De Luca et al. (2020)	This paper discussed the significant environmental impact of concrete production due to high emission of CO_2_	The findings of the paper indicate that recycling construction and demolition waste could yield a positive net benefit compared to producing virgin aggregate materials with similar properties. This approach not only offers financial advantages but also represents an environmentally sustainable option, requiring fewer resources for aggregate production
Ma et al. (2020)	This paper investigated challenges in CDW recycling in China through site visits to 10 recycling plants and interviews with 25 industry practitioners. Eight key challenges are identified, including lack of regulations	The study highlights the potential of recycling as an alternative to landfill for managing CDW in China but identifies significant challenges in policy implementation. Eight key challenges in CDW waste management identified in the study including unstable waste sources for recycling, lack of subsidies for recycling activities, inadequate focus on waste minimization in design etc
Tang et al. (2020)	This paper presented a review of studies focusing on recycling construction solid waste to produce geopolymer composites	Overall, the study highlights an economical and sustainable alternative for managing municipal and construction solid waste through recycling into construction materials such as geopolymer composite
Islam et al. (2019)	This study addressed the increasing CDW challenge in Bangladesh by estimating WGR and analyzing the economic benefits of recycling	Findings of the study reveal that recycling CDW waste presents entrepreneurial and economic opportunities, offering environmental benefits and significant CO_2_ emissions reductions
Turkyilmaz et al. (2019)	The paper focused on analyzing and improving CDW management practices in a leading construction company in Kazakhstan	The study mentions few recommendations including leveraging technologies like Building Information Modeling (BIM) for waste reduction and improving employee awareness through training programs. The findings of this study have implications for other construction companies in Central Asia as well
Whittaker et al. (2019)	A multi-step study done in the EU that was set up to develop sorting technologies to improve the quality of CDW-derived aggregate	The study reused CDW waste by repurposing concrete as aggregate and timber in new panels. Environmental impact analysis showed that the panels made of repurposed timber had close to 50% or a smaller number of environmental impacts when compared to virgin materials being used for construction

## Materials and method

### Study area

**Fig. 1:**
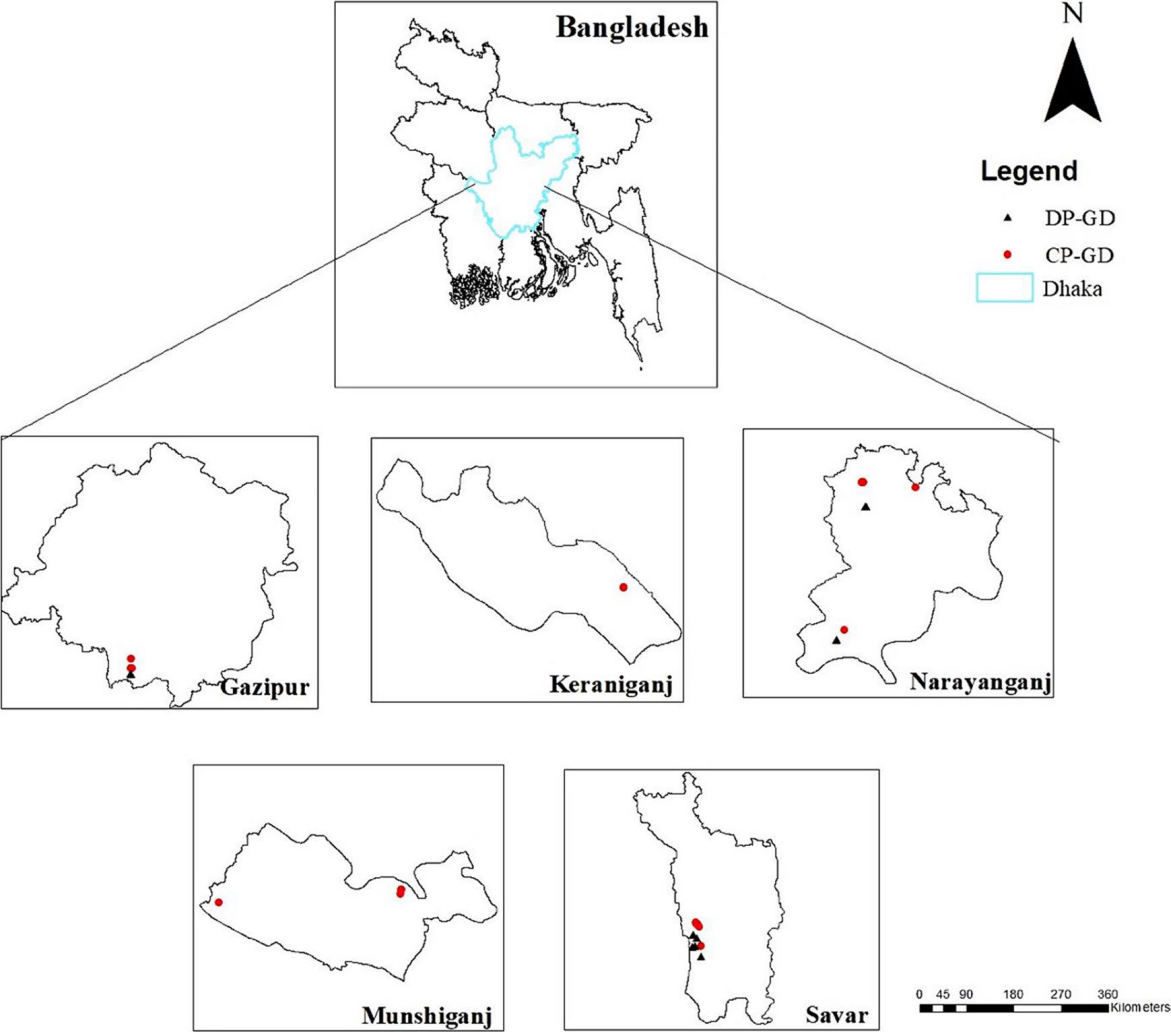
Map showing the location of the studied construction and demolition project sites within different regions of Greater Dhaka. Note: DP-GD – Demolition project site in Greater Dhaka; CP-GD – Construction project site in Greater Dhaka

In the past few decades, Dhaka City experienced rapid growth and is now one of the world’s largest megacities (Roy, 2021). In the 2001 census, the Dhaka Statistical Metropolitan Area covered a total of 1,353 km^2^, with Dhaka City Corporation occupying 276 km^2^. Currently, the metro area population of Dhaka is 23,210,000, which is a 3.26% increase from 2022 (Macrotrends, 2023). The metropolitan area includes the Dhaka City Corporation (north and south wing) referred herein as Dhaka City, as well as parts of neighboring areas such as Savar and Keraniganj. The study areas for the current project include Dhaka, Gazipur, Narayanganj, Manikganj, and Munshiganj located within Greater Dhaka region; covering an area of around 4929.45 km^2^ (Khan et al., 2024). Figure 1 shows the map of study areas focusing on the construction and demolition sites that were surveyed during the period of study (2022–2024).

### Details of the studied sites

For the present study, a total of 21 construction sites were surveyed within the study areas. Among the 21 construction project sites, four primary categories—residential (71.43%), mixed-use (residential and commercial) (19.05%), industrial/factory (4.76%), and commercial (4.76%)—were identified. A total of 12 demolition sites were considered that have a diverse occupancy type, incorporating residential (41.67%), mosque or religious infrastructure (8.33%), school (8.33%), industrial/factories (25.00%), reinforced cement concrete (RCC) water tank (8.33%), and mixed-use (residential and commercial) sites (8.34%). The selection of 21 construction and 12 demolition sites in Greater Dhaka was driven by a purposeful sampling technique (Palinkas et al., 2015), designed to encompass the variety of building and demolition activity in the area. This technique guaranteed the incorporation of diverse project kinds, dimensions, and developmental phases, enhancing the sample's representation within the region. The selected construction sites include residential, mixed-use, industrial, and commercial categories, whilst the demolition sites comprise residential, institutional, industrial, and mixed-use facilities. These diversified samples facilitate a thorough evaluation of CDW generation across multiple sectors and illustrate the CDW management situation in Greater Dhaka.

The residential lands or plots varied in size from 200.88 to 648.17 m^2^ and 267.84 to 468.72 m^2^ for the studied construction and demolition projects, respectively (Table 2). Demolished sites such as the mosque and school covered a land area of 364.60 and 30.04 m^2^, respectively. The studied demolished industrial facilities covered a land area ranging from 204.60 to 2673.71 m^2^ while the one industrial complex had an area of 32408.64 m^2^ within the studied construction projects. The number of floors within the construction sites ranged from 5—12. The floor area for mixed residential and commercial project sites varied from 222.81 to 736.56 m^2^ for both the construction and demolition projects.

**Table 2:** Key details of the studied construction and demolition projects within the Greater Dhaka area

Construction Projects (CP)						
Survey Date (dd.mm.yy)	Project Site ID	Location	Type	Status (during survey)	No. of Floors	Land Area (m^2^)
10.02.23	CP A	South Keraniganj	Residential	On going	G + 7	334.80
10.02.23	CP B	Keraniganj	Residential	On going	G + 8	334.80
17.02.23	CP C	Narayanganj	Industrial complex	On going	G + 1	32,408.64
20.03.23	CP D	Rupganj, Narayanganj	Residential	On going	G + 5	200.88
20.03.23	CP E	Rupganj, Narayanganj	Residential	On going	G + 7	334.80
20.03.23	CP F	Araihajar, Narayanganj	Mixed (commercial cum residential)	On going	G + 7	736.56
26.05.23	CP G	Gazipur	Residential	On going	G + 9	401.76
26.05.23	CP H	Gazipur	Residential	On going	G + 8	301.32
26.05.23	CP I	Gazipur	Residential	On going	G + 7	200.88
26.05.23	CP J	Gazipur	Residential	On going	G + 9	368.28
26.05.23	CP K	Gazipur	Residential	On going	G + 10	511.50
15.07.23	CP L	Savar	Residential	On going	G + 10	535.68
18.07.23	CP M	Sree Nagar, Munshiganj	Residential	On going	G + 2	234.36
11.08.23	CP N	Munshiganj	Commercial	On going	B + G + 10	669.60
11.08.23	CP O	Munshiganj	Residential	On going	G + 4	222.81
11.08.23	CP P	Munshiganj	Mixed (commercial cum residential)	On going	G + 9	405.11
11.08.23	CP Q	Munshiganj	Mixed (commercial cum residential)	On going	G + 7	222.81
19.08.23	CP R	Savar	Residential	On going	G + 9	648.17
19.08.23	CP S	Savar	Residential	On going	G + 7	648.17
19.08.23	CP T	Savar	Residential	On going	G + 9	405.11
19.08.23	CP U	Savar	Residential	On going	G + 7	303.83
Demolition Projects (DP)						
26.05.23	DP A	Gazipur	Residential	On going	Ground floor (tin shed)	468.72
26.05.23	DP B	Gazipur	Residential	On going	G + 5	401.76
15.07.23	DP C	Savar	Mosque	On going	Ground foor	364.60
15.07.23	DP D	Savar	Residential	On going	Ground floor (tin shed)	401.76
15.07.23	DP E	Savar	Residential	On going	G + 3	267.84
15.07.23	DP F	Savar	Residential	On going	Ground floor	334.80
18.07.23	DP G	Munshiganj	School	On going	G + 1	30.04
11.08.23	DP H	Narayanganj	Industrial (Textiles)	On going	G + 4	744.00
11.08.23	DP I	Narayanganj	Industrial (Textiles)	On going	Ground floor (tin shed)	204.60
15.08.23	DP J	Morapara, Rupganj	RCC water tank	On going	G + 2	202.55
15.08.23	DP K	Morapara, Rupganj	Industrial (Jute mill)	On going	Ground floor only	2673.71
19.08.23	DP L	Savar	Mixed (commercial cum residential)	On going	G + 1	648.17

*CP* – Construction project; *DP* – Demolition project; *G* – Ground floor; *B* – Basement; *RCC* – Reinforced cement concrete

### Identification of WGR and recycling potential

The study employed a primary qualitative survey based on a structured questionnaire (Appendix 1) and quantitative assessment through on-site visit within the studied construction and demolition project sites. Field data involved gathering information on CDW produced during the construction, rehabilitation, and demolition of buildings at site. Numerous methodological procedures, including photographic recording, and demarcation of geographic coordinates, were used to characterize the study location. Additionally, different techniques were used for data collection such as: direct observation of the composition of CDW, sorting and weighing waste items from a representative area, gravimetric analysis, and field observations regarding waste management and disposal techniques/issues.

The primary data obtained through such assessment were utilized to determine the WGR of the construction and demolition sites. The study mainly considered concrete, brick, cement, metal (i.e., steel rod), and mortar as the main waste components of the studied construction and demolition sites. For the WGR of the demolition and construction sites, the following Eqs. (1) and (2) were used, respectively. The waste components, including concrete, brick, metal, and mortar, were collected in separate sealed zip-lock, clear plastic bags from the studied construction and demolition sites. These samples were then transported to a controlled laboratory environment, where they were precisely weighed and analyzed to determine their mass composition within ~ 24 h after the field survey and stored at room temperature (~ 25 °C) for subsequent physio-chemical analyses. The volume of each waste component was estimated based on standard volume calculations from field measurements, and the waste composition was assessed through sample analysis of a representative waste dumping area within the studied construction and demolition sites followed by the methodology described by Lu et al. (2011). The density of the individual waste items was obtained from reference material properties and verified through third-party lab testing. A detailed methodology of the applied WGR calculation is further described by Haque et al. (2024).

1 $${\mathrm{WGR}}_{\mathrm{DP}}=\sum_{\mathrm{i}=1}^{\mathrm{N}}{\mathrm{WM}}_{\mathrm{i}}/\mathrm{FA}$$

2 $${\mathrm{WGR}}_{\mathrm{CP}}=\sum_{\mathrm{i}=1}^{\mathrm{N}}({\mathrm{WV}}_{\mathrm{i}}\times {\uprho }_{\mathrm{i}}\times {\mathrm{WC}}_{\mathrm{i}})/\mathrm{FA}$$

where,

WGR_DP_: Waste generation rate of the demolition projects (kg/m^2^);
WGR_CP_: Waste generation rate of the construction projects (kg/m^2^);
WM_i_: Mass of the i^th^ waste component (kg);
WV_i_: approximate volume of the i^th^ waste component (m^3^);
WC_i_: Composition of the i^th^ waste component (%);
ρ_i_: Density of the i^th^ waste component (kg/m^3^);
FA_i_: Representative floor area (m^2^).

Recycling CDW components into new construction and rehabilitation work can help in the reduction of natural resource consumption, energy usage and landfill space in addition to the economic benefits (Yazdani et al., 2021). It can also help in the business development and employment creation aspects for a nation. In Australia, the recycling rate of CDW was reported to be 66.80% and in Singapore, this rate was found to be 99% for the concrete and masonry waste (Villoria-Sáez et al., 2020). Only in Queensland, the recycling rate was 50% in 2015 (Tuladhar et al., 2020). To identify the recycling potential of the generated CDW within the Greater Dhaka this study employed Eq. (3) to calculate the recycled amount of the studied waste components (Islam et al., 2019).

1 $${\mathrm{RE}}_{\mathrm{i}}={\mathrm{WGR}}_{\mathrm{i}}\times {\mathrm{WC}}_{\mathrm{i}}\times {\mathrm{R}}_{{\mathrm{CDW}}_{\mathrm{i}}}$$

where,

$${\mathrm{RE}}_{\mathrm{i}}$$
The maximum recycled amount of the i^th^ CDW material (kg/m^2^);
$${\mathrm{WGR}}_{\mathrm{i}}$$
The waste generation rate of the i^th^ CDW material from both construction and demolition projects (kg/m^2^);
$${\mathrm{WC}}_{\mathrm{i}}$$
The waste composition rate of the i^th^ CDW material based on this study (%);
$${\mathrm{R}}_{{\mathrm{CDW}}_{\mathrm{i}}}$$
The maximum recycling rate for the i^th^ CDW material (%).

### Physio-chemical analysis

The CDW samples collected from the studied sites were used for Gravimetric Analysis. As the collected samples were commingled, the analysis was performed following a series of tasks. First, the samples were segregated and labeled according to the site codes. Then, the mortar was segregated from concrete and brick samples using a chisel and hammer carefully to avoid any sort of loss. Next, appropriate quantities of these samples were crushed using a crusher. The crushed and segregated samples were carefully weighed by using a mass balance. The volume of the sample was obtained by using the dimensions of a cuboidal container used for experimental purposes. Finally, these data were utilized to calculate the density and unit weight of the samples to understand their gravimetric composition. Additionally, the study employed a chemical characterization (pH and heavy metals) on waste brick and mortar samples taken from different construction and demolition sites which will enable to identify any human risk associated with the recycling of CDW (Table 3). Previous studies have shown that heavy metals can leach into the soil, posing environmental risks. For instance, studies conducted by Wu et al. (2022), and Balali et al. (2023) found that Cd poses the highest risk to the soil environment. Studies from Ullah et al. (2023) highlighted the effects of Cr and Landes et al. (2023) found Pb as major contaminants within soil from construction materials. Additionally, a significant correlation exists between soil pH and heavy metal levels as stated by Diotti et al. (2021).

**Table 3:** Analyzed chemical parameters on the selected CDW samples

Parameters	Method of analysis	Instrumental method, brand and model of the equipment
pH	USEPA 150.1; SM 4500-H + B	pH meter, WTW, German
Cadmium (Cd)	USEPA 213.2; SM 3113 B	Atomic Absorption Spectophotometer, Shimadzu AA 7000, Japan
Lead (Pb)	USEPA 200.9 REV. 2.2; SM 3111 B	Atomic Absorption Spectophotometer, Shimadzu AA 7000, Japan
Chromium (Cr)	SM 3113 B	Atomic Absorption Spectophotometer, Shimadzu AA 7000, Japan

Calibration standards from HACH, USA, and SHIMADZU, JAPAN are used for standardization. To ensure QA/QC, the instructions stated in the test parameters standard procedure are followed

## Results

### Waste generation rate

**Fig. 2:**
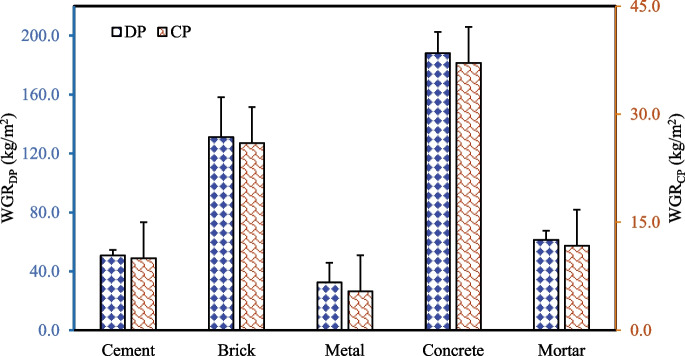
The waste generation rates of the studied construction and demolition projects of the Greater Dhaka region (2022–2024)

Figure 2 illustrates the average WGR of different CDW components from the 21 construction and 12 demolition projects of different parts of the Greater Dhaka Region. Since each construction project has a different design and occupancy purpose, the material requirements were also different. Hence, the WGR of construction as well as the demolition sites varied from site to site (Kitchenham & Pfleeger, 2002). The total WGR for the demolition projects (DP) was found to be approximately 463.67 kg/m^2^, where 40.57% of waste is from the concrete followed by brick (28.28%), mortar (13.23%) and cement (10.93%). On the other hand, the WGR from the construction projects was calculated to be 90.31 kg/m^2^ of which 41.12% is contributed by the concrete followed by brick (28.79%) and mortar (13.01%). The variation in the data suggests that the WGR is dependent on various factors such as construction type, building size, construction pattern, and measurement approach, among other factors (Parisi Kern et al., 2015; Wang et al., 2020). Arshad et al. (2017) reported that bricks (6.82%), tiles (6.68%), plaster (6.63%), and wood (6.41%) are the major waste components in the construction sector of Pakistan where project management followed by the handling of material are the major factors in the waste generation. In the Bengaluru City of India, the WGR of construction and demolition activities were reported to be 5.72 and 57.55 kg/m^2^, respectively (Abhishek & Joshi, 2022) and in Mumbai, the total CDW amount was around 0.91MT in 2022 (Swetha et al., 2022). In Chile, the WGR was reported to be 186 kg for per m^2^ of constructed floor area by Bravo et al. (2019).

The northern region of Dhaka city is expanding due to the concentration of several industrial and residential zones starting from Mirpur, and Ashulia, to Tongi, Gazipur, Narayanganj, Rupganj and Konabari. The industries in the areas of Aminbazar, Savar, Nabinagar, and Manikganj are expanding in the northwest. The construction of private universities in the regions of Purbachal, Ashulia, Bashundhara, Savar, and Gazipur is accelerating urban expansion. According to Karim (2021), between 2000 and 2010, the urban area around Dhaka grew from 79.74 km^2^ to 99.23 km^2^, marking a net increase of 6.39%. Similarly, from 2010 to 2020, Dhaka's urban area increased from 99.23 km^2^ to 133.52 km^2^, reflecting a net growth of 11.25%. Wang and Sarker (2020) analyzed the built-up area increment rate within the Greater Dhaka from 1990 to 2017 considering its rapid and ever-growing residential and industrial development. Based on these reported results the present study projected built-up land area from 2018 to 2030 for the major eight districts – Dhaka City, Gazipur, Savar, Narayanganj, Keraniganj, Bandar, Rupganj and Sonargaon – of the Greater Dhaka Region (Appendix 2). Based on the change in land use pattern and built-up land area, the current study further calculated the total construction and demolition floor area for these eight districts considering a yearly growth rate of real estate sector as 8% for the whole nation (Appendix 3). Considering the average WGR of 463.67 kg/m^2^ and 90.31 kg/m^2^ for the construction and demolition projects of the Greater Dhaka Region, the present study also evaluated the WGR of the total CDW in these eight districts from years 2022 to 2030 (Table 4). It has been found that the highest increase in terms of CDW will be at Savar following its highest land area conversion to industrial and residential hubs within the Greater Dhaka followed by Rupganj. As indicated earlier that the built-up area is expanding to the surrounding parts of the Dhaka city, the current analysis also confirms that the CDW amount will be growing proportionally within the region in this timeframe.

**Table 4:** Projected increase in the CDW in the major eight districts of the Greater Dhaka area from 2022 to 2030

	Construction waste (MT)								
	2022	2023	2024	2025	2026	2027	2028	2029	2030
Dhaka city	0.34	0.36	0.39	0.42	0.46	0.49	0.53	0.58	0.62
Gazipur	0.59	0.80	1.12	1.60	2.34	3.45	5.14	7.71	11.60
Savar	1.50	2.92	5.89	12.05	24.87	51.54	107.01	222.40	462.40
Narayanganj	0.42	0.55	0.73	1.02	1.45	2.09	3.07	4.55	6.79
Keraniganj	0.31	0.37	0.48	0.64	0.89	1.28	1.88	2.81	4.24
Bandar	0.30	0.37	0.49	0.68	0.98	1.47	2.26	3.53	5.56
Rupganj	0.43	0.65	1.06	1.84	3.32	6.11	11.38	21.35	40.18
Sonargaon	0.30	0.41	0.60	0.96	1.63	2.85	5.12	9.31	17.05
	Demolition waste (MT)								
	2022	2023	2024	2025	2026	2027	2028	2029	2030
Dhaka city	0.52	0.56	0.60	0.65	0.70	0.76	0.82	0.88	0.95
Gazipur	0.87	1.19	1.67	2.41	3.53	5.23	7.81	11.72	17.65
Savar	2.26	4.43	8.94	18.34	37.88	78.53	163.10	339.00	704.80
Narayanganj	0.62	0.81	1.09	1.52	2.18	3.16	4.65	6.91	10.32
Keraniganj	0.44	0.54	0.70	0.95	1.33	1.92	2.84	4.25	6.43
Bandar	0.43	0.54	0.72	1.01	1.47	2.22	3.42	5.35	8.45
Rupganj	0.62	0.95	1.59	2.78	5.03	9.28	17.32	32.51	61.21
Sonargaon	0.44	0.60	0.89	1.44	2.45	4.32	7.78	14.16	25.97

*MT* – million tonnes

### Physio-chemical properties

Table 5 shows the results of the density of the selected CDW materials collected from the studied 15 construction and 12 demolition sites. From the gravimetric analysis, the mean density values of brick, mortar and RCC were found to be 1691.69 kg/m^3^, 2029.80 kg/m^3^, and 2357.55 kg/m^3^, respectively. The waste brick, mortar, and RCC from the demolition projects have average density values of 1525.16 kg/m^3^, 1832.99 kg/m^3^, and 2161.74 kg/m^3^, respectively.

**Table 5:** Gravimetric analysis results of the selected waste materials from the studied construction and demolition sites

Density of Construction Waste (kg/m^3^)			
Sampling Sites	Brick	Mortar	RCC
CP G	1384.21	1869.66	-
CP H	1465.20	1964.29	-
CP I	1696.83	2121.91	-
CP J	1925.13	2308.27	-
CP K	1801.80	2289.67	2346.81
CP L	1487.64	2233.85	-
CP M	1680.49	1923.08	2418.15
CP N	2034.78	2048.01	-
CP O	1686.91	1838.24	2188.55
CP P	1795.74	2091.13	2523.49
CP Q	1527.47	2075.47	2395.47
CP R	1920.73	1862.37	2278.07
CP S	1762.52	2010.94	2353.59
CP T	1449.28	1815.15	2306.44
CP U	1756.64	1994.95	2407.41
Avg	1691.69	2029.80	2357.55
Std. error	50.25	41.12	31.79
Density of Demolition Waste (kg/m^3^)			
DP A	1525.55	1854.04	-
DP B	1498.72	1707.21	-
DP C	1512.10	1757.86	1959.75
DP D	1493.93	2266.01	2574.77
DP E	1731.60	1906.32	2487.56
DP F	1753.16	2309.88	2292.77
DP G	1757.98	2006.17	2481.39
DP H	1616.03	2198.89	2400.96
DP I	1699.01	1981.87	2477.48
DP J	1863.14	-	2261.16
DP K	1611.11	-	2268.86
DP L	1547.99	1936.66	2346.84
Avg	1525.16	1832.99	2161.74
Std. error	117.54	171.64	198.95

The pH value of brick-and-mortar samples collected from both construction and demolition sites mostly ranges from 6.50—10.0 (Table 6). The mean pH value of waste brick samples from construction sites was found to be 7.56 whereas the mean pH value is 7.70 for waste brick samples at the demolition sites. However, the mean pH value for mortar samples is 8.67 at the construction sites which has reduced to 7.92 at the demolition sites.

**Table 6:** The pH value of the commonly found construction and demolition waste materials from different studied sites

Construction Waste		
Sampling Site	Brick	Mortar
CP A	6.50	10.00
CP B	-	-
CP C	7.00	10.00
CP D	-	-
CP E	7.00	7.00
CP F	-	-
CP G	8.00	8.50
CP H	7.50	9.00
CP I	8.00	8.00
CP J	8.00	9.00
CP K	8.00	8.00
CP L	7.50	7.50
CP M	8.00	9.00
CP N	7.00	7.00
CP O	8.00	8.50
CP P	7.00	9.00
CP Q	8.00	8.50
CP R	8.00	10.00
CP S	7.50	9.00
CP T	7.50	10.00
CP U	7.50	8.00
Avg	7.56	8.67
Std. error	0.11	0.23
Demolition Waste		
Sampling Site	Brick	Mortar
DP A	7.50	9.00
DP B	8.00	7.00
DP C	8.00	7.00
DP D	7.00	7.00
DP E	7.50	7.50
DP F	8.00	7.50
DP G	8.00	10.00
DP H	8.00	7.50
DP I	8.00	8.00
DP J	-	10.00
DP K	-	7.50
DP L	7.00	7.00
Avg	7.70	7.92
Std. error	0.13	0.32

### Environmental implications of heavy metal

The representative heavy metal content analysis from both construction and demolition waste samples is summarized in Table 7. The Pb concentration in mortar samples from construction sites ranges from 0.179 to 0.236 mg/L whereas at demolition sites this concentration ranges from 0.160 to 0.189 mg/L. The Pb concentration level was found to be much lower than the recommended level of US EPA (5.0 mg/L). Also, the Cd concentration levels in the analyzed samples did not exceed the maximum level by US EPA (1.0 mg/L). In the waste mortar sample from sampling site CP R (Table 5), the Cr concentration is found to be 0.089 mg/L which does not exceed the US EPA standard of Cr in soil (5.0 mg/L). In Skopje City (in the Republic of Macedonia), the Cr concentrations were found to be higher in the majority of the CDW samples than the recommended level by the Italian standard (Bianchini et al., 2020). Based on the heavy metal analysis results of this current study, the recycling of CDW material will be safe in terms of their health implications in the Greater Dhaka region.

**Table 7:** The analyzed heavy metal content test results of selected construction and demolition projects

Analyzed Metal Content	Construction Projects				Demolition Projects				Minimum Detection Level (MDL)	US EPA Guideline Value	Analytical Method
	CP I (Brick)	CP I (Mortar)	CP R (Brick)	CP R (Mortar)	DP B (Brick)	DP B (Mortar)	DP F (Brick)	DP F (Mortar)			
	mg/L	mg/L	mg/L	mg/L	mg/L	mg/L	mg/L	mg/L	mg/L		
Lead (Pb)	< MDL	0.236	< MDL	0.179	< MDL	0.160	0.151	0.189	0.010	5.00	USEPA 200.9 Rev 2.2; SM 3111 B
Cadmium (Cd)	< MDL	< MDL	< MDL	< MDL	< MDL	< MDL	< MDL	< MDL	0.001	1.00	USEPA 213.2; SM 3113 B
Chromium (Cr)	< MDL	< MDL	< MDL	0.089	< MDL	< MDL	0.007	< MDL	0.001	5.00	USEPA 200.9 Rev 2.2; SM 3111 B

### Recycling potential identification

**Fig. 3:**
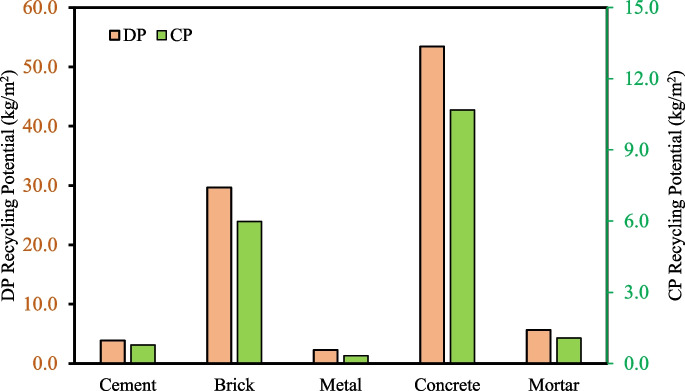
Recycling potential of the commonly found CDW materials in the Greater Dhaka region

It is well-established that approximately 70% of concrete and mortar waste can be recycled by converting into aggregates (Letelier et al., 2017; Mohammed et al., 2019), whereas for bricks and metals, 80% and 100% recycling can be achieved, respectively (Zhu & Zhu, 2020). Considering these values to be the optimum recycling rates, the present study identified the recycling potential of the most found CDW materials – cement, brick, metal, concrete, and mortar—for construction and demolition projects in the context of the Greater Dhaka Region (Fig. 3). For per m^2^ demolition activities, 53.41 kg of concrete waste can be recycled followed by 29.67 kg of brick waste for further usage. For construction projects, the recycling potential of concrete and brick waste materials is 10.69 kg/m^2^ and 5.99 kg/m^2^, respectively. A statistically significant correlation (r = 0.97; p < 0.05) is observed between the construction and demolition waste generation rate and its recycling potential.

### Analysis of economic benefits

**Fig. 4:**
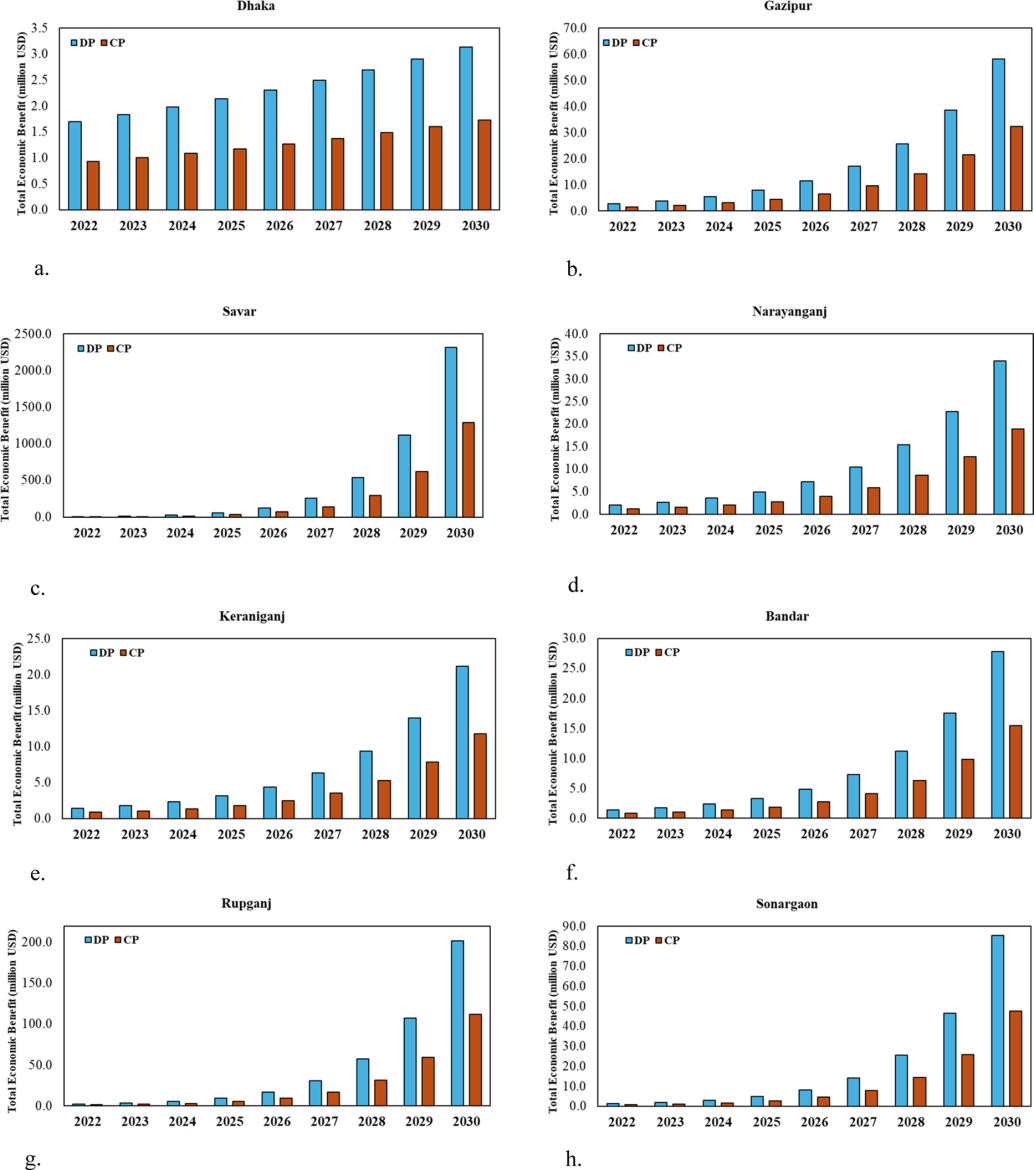
Total economic benefits from recycling waste materials from both demolition and construction projects—**a**. Dhaka, **b**. Gazipur, **c**. Savar, **d**. Narayanganj, **e**. Keraniganj, **f**. Bandar, **g**. Rupganj and **h**. Sonargaon – in the studied regions of Greater Dhaka from the years 2022 to 2030. Note: 1.00 USD = 117.20 BDT (as of Jul 06, 2024; Source: https://www.forbes.com/advisor/money-transfer/currency-converter/usd-bdt/)

In the present study, an economic benefit analysis was conducted considering the growth of the construction and demolition footprint in the study areas between 2022 and 2030. The identified recycling potential (RE_i_) values for each of the CDW items were used to calculate their recycled amount. Following that, the recycling values of those CDW materials were used to calculate the financial outcome that can be expected by recycling the CDW materials for the studied years. The recycling value of the studied CDW items was collected through a field-based survey at different salvage yards in Dhaka City as given in Appendix 4. The total economic benefits of the waste from both construction and demolition projects were assessed based on the combined values of the studied waste materials for the respective years. For instance, Savar exhibits the greatest potential for economic benefits, projected to reach 2319.54 million USD by 2030 for DP and 1287.71 million USD for CP, significantly exceeding the other cities studied (Fig. 4c). Rupganj demonstrates significant potential, projecting 201.47 million USD for the demolition and 111.89 million USD for construction projects by 2030. Dhaka City, although less than Savar and Rupganj, demonstrates significant potential, with projected values of 3.14 million USD for DP and 1.73 million USD for CP by 2030. In 2025, the DP and CP of Savar are projected to generate 60.36 million USD and 33.56 million USD, respectively, whereas Rupganj's DP and CP are expected to yield 9.14 million USD and 5.13 million USD, respectively. By 2027, the demolition and construction projects of Savar may reach 258.45 million USD and 143.53 million USD, respectively, whereas Rupganj's DP and CP could amount to 30.55 million USD and 17.01 million USD, respectively. Gazipur, Narayanganj, Keraniganj, Bandar, and Sonargaon exhibit potential for economic benefits, although to a lesser degree than the other regions investigated.

The economic benefit from recycling metal waste from demolition projects is recorded to reach around 947,670 USD by 2030 in Dhaka (Appendix 5: Figure 7) whereas in Narayanganj this figure will be around 21.52 million USD. Similarly, the economic benefit is recorded for the construction projects (Appendix 5: Figure 8). By 2030, the economic benefits achieved by recycling brick and concrete waste into aggregates from the construction projects within Keraniganj are around 6.52 million USD and 803,950 USD, respectively. In Rupganj, the recycling and reusing of brick and concrete from the constructed floor areas into new construction projects will result in savings of around 41.37 million USD and 7.63 million USD by 2030, respectively. A significant increase in the economic benefit also confirms the fact that the urban sprawl is growing at a substantially faster rate within the adjacent districts of Dhaka city due to the change in land cover and rapid growth in the construction and demolition activities.

## Discussion

### Environmental implications and management

In Bangladesh, the Building Construction Regulations 2008 and the Bangladesh National Building Code 2020 encourage sustainable construction practices (BNBC, 2020). The environmental implications of CDW are becoming a pressing issue in urban waste management. However, no legislation encourages the use of recycled CDW in new construction and rehabilitation projects. UNEP (2015) indicated that CDW is a large source of total solid waste generation (estimated at 36% by weight) worldwide but receives significantly less attention in terms of its management. This is partially explained by the fact that the components of CDW are harmless but largely differentiated as relatively high-density materials (Cook et al., 2022). Furthermore, as waste is typically produced during normal business hours, a large portion of the detrimental effects on the environment or public health occur behind the public view (Cook et al., 2022).

Several studies found that CDW is a serious environmental concern and has long-term consequences such as increasing pollution, resource depletion, and land deterioration (Ding et al., 2023; Islam et al., 2019). According to Islam et al. (2019), despite a lack of evidence, there is a strong indication that the combustible fraction of construction and demolition waste is disposed of by open burning in many low- and middle-income countries, including increasing amounts of high chloride-content PVC, putting people at risk of exposure to dioxins and other compounds. The findings of the present study indicate that unsafe working conditions at the CDW sites pose serious hazards to both the workers and the environment.

In Bangladesh as naturally occurring stone aggregates are hard to find, clay-burnt bricks are used as construction material. Approximately 7000 brick kilns, including 1000 near Dhaka, are privately operated in the country to meet the demand for bricks (Haque et al., 2022). The brick manufacturing industry is anticipated to grow at a pace of 6% per year during the next ten years (WB, 2012). Previous studies have highlighted the air pollution issues connected with the brick (Haque, 2019; Haque & Sharif, 2021) and cement manufacturing (Nayeem et al., 2022; Rahman et al., 2015) processes as well as the soil pollution from particulate emissions within the Greater Dhaka area (Haque et al., 2022). Specifically, in Dhaka, many workers are classed as informal, resulting in an unregulated and vulnerable workforce who are at high risk of exposure to hazards. An efficient management and recycling practice of CDW will help to lower the negative environmental issues of this region.

### Waste management and recycling

For efficient waste management, infrastructure development, and relevant policy formulation, waste quantification is the fundamental step (Akhtar & Sarmah, 2018; Haque et al., 2021). Through extensive fieldwork, this study has computed the CDW generation rate within the Greater Dhaka area and projected WGR from 2022 to 2030 considering the changes in land cover and usage over the years. The findings suggest that large quantities of concrete waste (~ 41%) are produced during construction and demolition activities, which is one of the most widely used construction materials. The concrete production process contributes to carbon emissions and waste generation. Concrete recycling involves crushing and reusing concrete waste instead of disposing of it in landfills. This circular strategy is expected to curb both waste generation and carbon emissions while protecting natural resources (Küpfer et al., 2023). Recycling other CDW materials can also lower construction costs and create job opportunities in the construction and recycling industries. In this regard, the recycling potential identified for each of the waste components in this study is expected to aid in deriving relevant policy measures to establish an efficient CDW recycling industry can be established.

Recycling CDW is not a new concept, and reclaimed concrete, mortar, and bricks from the crushing process are utilized as a road foundation in road structures all over the world, including China, Japan, Turkey, many European countries, and the United States (Akbas et al., 2020; Aurstad et al., 2006; Li, 2008; Mahdi, 2017). Recycling rates are found to be 47% (in France), 75% (in Italy), more than 65% (in Germany and Belgium), and 86% (in the UK) (Zheng et al., 2017). For instance, reclaimed concrete works effectively as a filler in sound-absorbing embankments and as a material for cut-off layers, main foundations, and auxiliary foundations (Stępień & Maciejewski, 2022). The usage of reclaimed concrete and mortar aggregates in various elements of structural applications has been demonstrated to be effective with some types of adjustments in their mix design and other improvements (Xiao et al., 2022, Nwakaire et al., 2020, Rahaman et al. (2020) studied that the shortage of road construction materials in Bangladesh and found that pavement recycling has not yet become widespread, thus construction and maintenance rely heavily on imported virgin construction materials, although recycled CDW materials have been used in the pavement construction for many decades in industrialized countries.

**Fig. 5:**
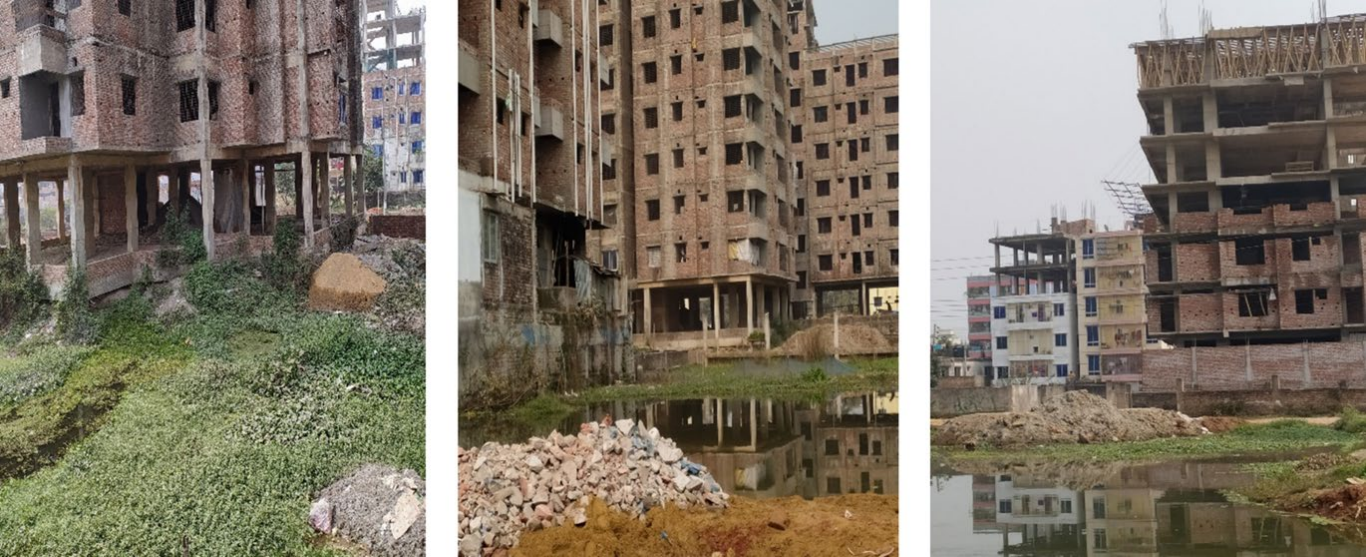
Wetlands being filled with CDW and sand by private developers to construct residential and commercial plots and infrastructures (from different parts of the Greater Dhaka area)

In the Greater Dhaka area, CDW is typically disposed of in designated landfill sites—Matuail (North Dhaka) and Aminbazar (South Dhaka)—and other open spaces depending on the location of construction and demolition sites. Findings reveal that numerous plans are being considered regarding Dhaka city's eastward expansion across the Balu and Sitalakshya rivers (Roy et al., 2019; Siddiqui, 2024). As of January 2024, field observation indicates that many areas of this region are already being developed rapidly and haphazardly at an alarmingly rapid pace. Private developers are buying property and filling up low-lying flood plains, rivers, canals, and other water bodies with sand and CDW to build and sell new residential and commercial plots (Fig. 5). Other viable options for the city’s horizontal growth include expanding across the Buriganga River to Keraniganj and beyond to Mawa, or to the southwest across the Padma River to Shariatpur and Madaripur, supported by new infrastructure developments such as the newly constructed Padma Bridge, a multipurpose road-rail bridge that is also one of the largest infrastructure projects in the country’s history (Rashid et al., 2023). In the future, to achieve sustainability, in addition to residential and commercial properties, highway and bridge construction projects can explore the use of recycled materials instead of imported alternatives (Martínez-Muñoz et al., 2021). Furthermore, the authors suggest that in-place recycling-repaving of aggregates used in road rehabilitation and maintenance works in the country can be a practical solution to ongoing and future material demand.

**Fig. 6:**
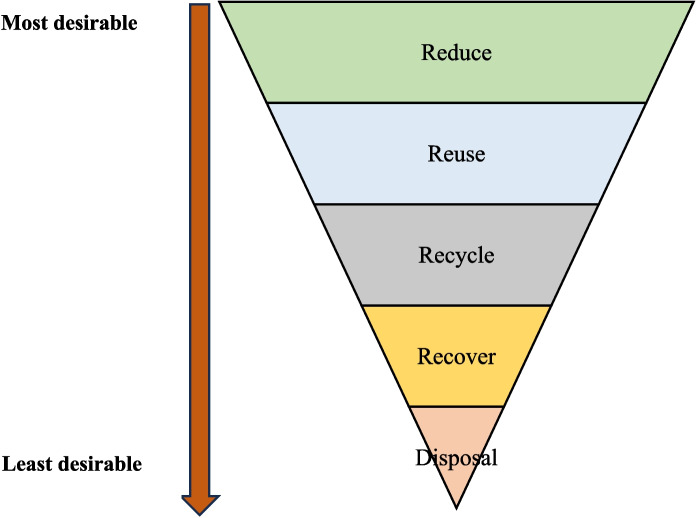
The waste management hierarchy (adapted after Purchase et al., 2022)

**Fig. 7:**
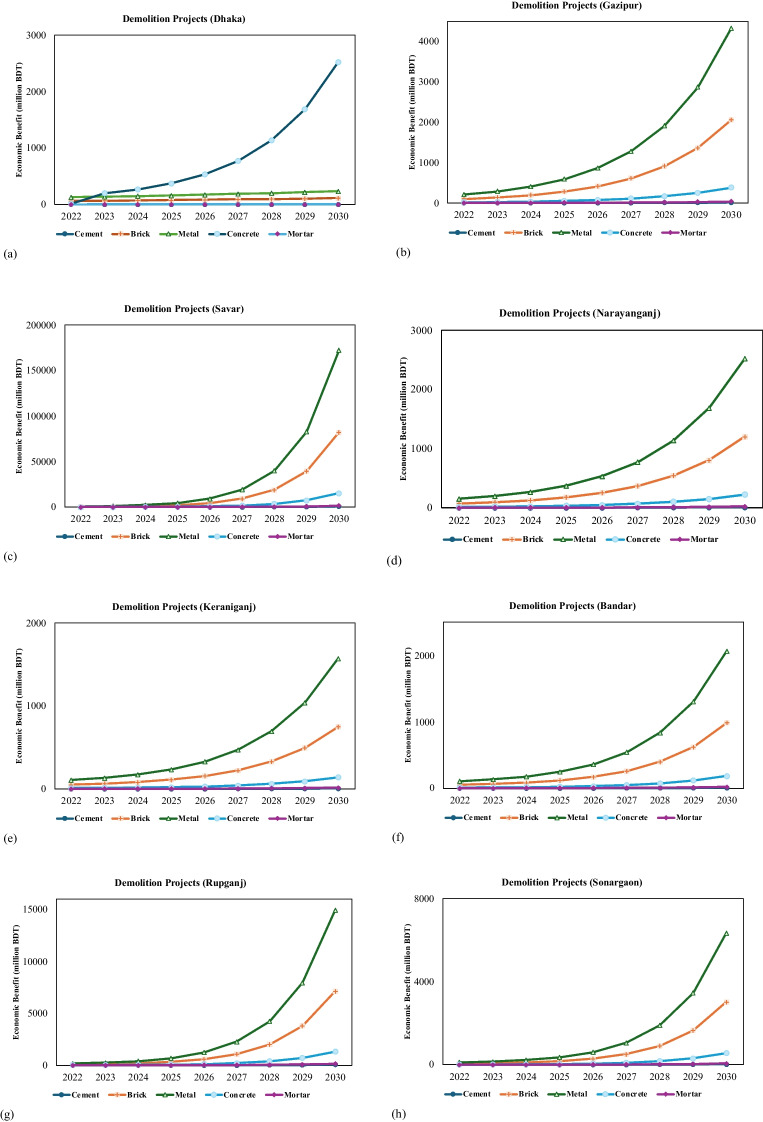
Economic benefit analysis from recycling CDW from demolition projects in the Greater Dhaka Region –**a** Dhaka; **b** Gazipur; **c** Savar; **d** Narayanganj; **e** Keraniganj; **f** Bandar; **g** Rupganj; **h** Sonargaon. Note: 1.00 USD = 117.20 BDT (as of Jul 06, 2024; Source: https://www.forbes.com/advisor/money-transfer/currency-converter/usd-bdt/)

**Fig. 8:**
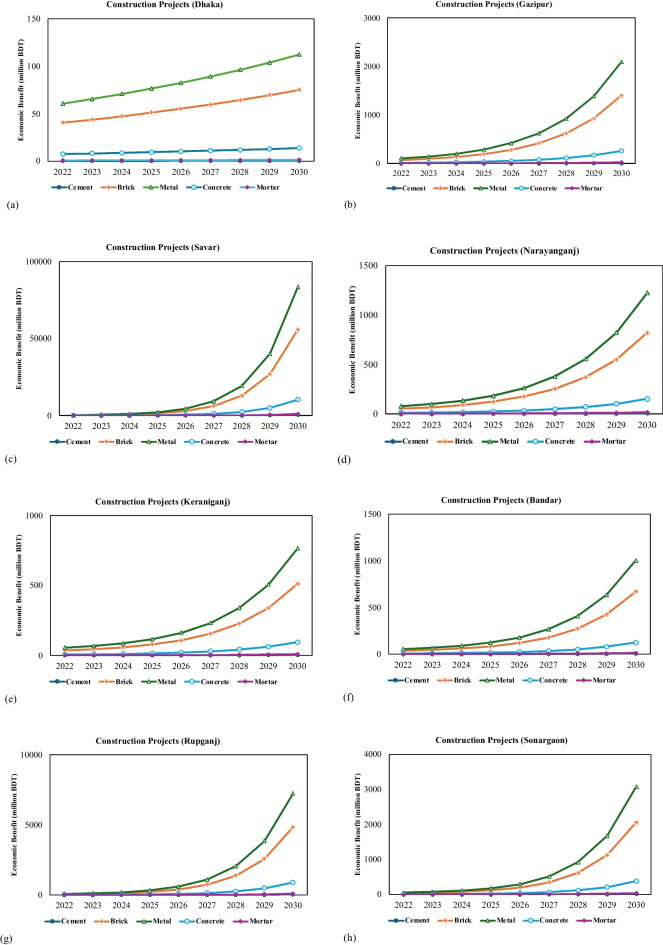
Economic benefit analysis from recycling CDW from construction projects in the Greater Dhaka Region –**a** Dhaka; **b** Gazipur; **c** Savar; **d** Narayanganj; **e** Keraniganj; **f** Bandar; **g** Rupganj; **h** Sonargaon. Note: 1.00 USD = 117.20 BDT (as of Jul 06, 2024; Source: https://www.forbes.com/advisor/money-transfer/currency-converter/usd-bdt/)

In both developed and developing countries, the waste management hierarchy based on the 3R principle (Fig. 6) provides the foundation for the circular economy approach within the construction industries (Ogunmakinde et al., 2022). Through the 3R approach, the industry’s environmental sustainability is enhanced by reducing natural resource consumption by recycling construction materials and minimizing the waste loads from getting into landfills (Guerra et al., 2019; Suthar et al., 2016). Table 8 shows the best management practices that can be achieved in the context of Bangladesh for efficient CDW management in different stages of construction activities.

**Table 8:** The best management practices that can be achieved in different stages of the construction activities

Stages	Causes of waste	Best management practices
Design	Design alteration, design and detailing errors, miscommunication of client and real estate, unclear specifications, etc	• The design stage should have the flexibility to accommodate future design alterations; • Building shape and form should be selected in such a way that it reduces the use of excess construction material and generates minimal waste
Procurement	Ordering errors, supplier errors, over allowances (difficulty in ordering a product in small quantity)	• Awareness build-up training and educational programs for the construction workforce about efficient ways to minimize waste generation
Transportation	Inefficient loading and unloading methods, damage during transportation, difficulty in accessing construction sites	• Usage of selective building demolition plans and supervision of the proper implementation of the waste sorting and transportation plans
On-site management and planning	Lack of and improper onsite management plans, lack of supervision, improper planning for the required quantity of construction material	• Identification of all potential waste and development of specific actions for every type of waste produced, including their reuse and recycling opportunities; • Segregation and handling of mono-fractional waste streams whenever feasible; • Monitoring both on- and off-site waste management practices and the promotion of adequate communication between key stakeholders for efficient CDW management operations
Material storage and handling	Improper and insufficient site storage, materials stored at a considerable distance from the point of application, materials supplied in loose form (bricks, sand, glass)	• Encouragement of appropriate material handling and storage procedures to prevent material loss through appropriate logistics; • Planning of supply chain management effectively to control material stocks
Site operation	Unused materials and products, equipment malfunction, poor craftsmanship, use of wrong materials resulting in their disposal, time pressure	• Encouragement of off-site construction, employment of prefabricated components, precast beams, contemporary building techniques, and the rental and reuse of auxiliary equipment; • Maximising material recovery at the end of its useful life: use of fewer distinct materials and parts; • Use of materials that are simple to separate and standard size
Waste/Residual	Packaging, over-preparation of materials, off-cuts from cutting materials to a specific length/shape	• Ensuring proper usage and maintenance of technical equipment required in waste sorting, transport, and disposal operations; • Awareness building programs among key stakeholders about the economic and environmental benefits of construction and demolition waste valorization approach

Source: based on Oliveira et al. (2021); Esa et al. (2017); Fatemi (2012)

### Business development, policy implications, and achieving sustainable development goals (SDGs)

As Bangladesh is progressing toward its graduation from the United Nations’ list of Least Developed Countries by 2026, there is large potential for CDW management. Effective CDW management and recycling practices can bring significant environmental, economic, and social benefits. The recycling potential and economic benefits associated with the studied waste components of this study will help to devise the public–private partnership (PPP) that can lead to the establishment of large-scale recycling facilities to enhance CDW management infrastructure. Key actors in this field include facility owners, contractors, demolition companies, recycling firms, and all the concerned persons contributing to the shared goal of minimizing waste and promoting recycling. In Sweden, there’s been progress in establishing new rules for CDW management driven by environmental managers within large construction and demolition companies with the help of the government. Their efforts have led to collaborative projects aimed at overcoming recycling barriers (Andersson & Buser, 2022). It's essential to implement well-designed penalties, subsidies, and financial support policies to ensure consumer demand for recycled products that influence the evolution of CDW management facilities. Penalties are effective in curbing the behaviors of non-cooperative recyclers and increasing the availability of recycled products in the market (Lu et al., 2022).

Another approach to achieve better CDW management (which particularly requires a policy-level mechanism) is adopting a Circular Economy (CE) approach. Originating from ideas by Boulding (1966) and Pearce and Turner (1990), CE aims to preserve natural resources, maximize material value, and design production systems for closed-loop material and energy flows. This approach is encouraged by the principles of the 3R and supported by concepts such as Industrial Ecology and regenerative design (Oliveira et al., 2021). The promotion of the CE business model can result in economic and environmental benefits, including resource conservation, waste valorization, job creation, reduced energy consumption, and decreased greenhouse gas emissions. This also enhances business and community resilience by reducing reliance on long-distance supply chains. In developing countries, the adoption of the CE model is still in the primary stage. With improved policies and laws, strategies that combine social aspects, establish a market for CDW reuse and recycling, ensure safe disposal, and enforce strict supervision can positively transform CDW management in Bangladesh through the CE approach. Moreover, digitization tools (i.e., Building information modelling, green building practices, digital twins, GIS, cloud-based waste tracking and on-site management, sustainable supply chain management, and automated construction system) play a key role in the construction industry's efforts (Daoud et al., 2020) to achieve zero waste and CE by recovering, reusing, and recycling material and enable businesses to easily monitor the number of materials utilized on each project, allowing for more effective resource management. In Bangladesh, the adoption of digital technology was propelled by the government’s determined attempts to meet national and sustainable development objectives. Real estate development companies in Dhaka have long included digitalization; new building construction already makes extensive use of different types of software, which can generate visualizations such as computer-aided design and BIM. The local CDW industries can incorporate various construction management software to streamline reuse and recycling processes to optimize resource utilization. For example, digitalization tools can provide real-time, secure access to centrally managed data to project participants which lowers the risk of errors and improves project management and collaboration. Additionally, access to affordable digital technology (i.e., a national database regarding the waste composition, volume, and recycling potential from projects), can allow workers to minimize the environmental impact of CDW while making more informed decisions. A more specific and actionable policy recommendation for the development of sustainable construction practices and the establishment of recycling facilities for CDW management in Greater Dhaka has been summarized in Table 9.

**Table 9:** Identification of barriers and actionable measures for the efficient CDW management in the Greater Dhaka area by the responsible agencies

Challenges/Limitations	Proposed Recommendations	Responsible Agencies
*Regulatory and political* • Lack of strict enforcement of waste management policies and regulations in Bangladesh • Absence of mandatory requirements for recycling of waste in construction and demolition projects • Limited government intervention and enforcement in promoting sustainable construction practices • Bureaucratic challenges and lack of political awareness in establishing recycling facilities and integrating CDW into actionable waste management plans	*Legislative measures to promote the CDW recycling* • Introduction of legal requirements for construction companies to recycle a minimum percentage of CDW materials in new development projects • Enforcement of legislative measures where developers and contractors are accountable for the proper disposal and recycling of waste generated during construction and demolition activities • Implementation of stricter guidelines on landfill disposal to discourage illegal dumping and promotion of recycling practices	DoE, PWD, City management authorities (i.e., RAJUK), and LGED
*Financial* • High initial investment costs for the establishment of CDW recycling plants and infrastructure • Limited resources or access to government funding for businesses that promote recycled materials • Lack of market demand for recycled construction materials, discouraging private sector investment • Higher costs of processing recycled materials compared to sourcing virgin materials and ignoring environmental issues associated with those items	*Financial incentives and market development* • Promotion of financial incentives for private companies that use recycled construction materials or investment in CDW recycling infrastructures • Encouragement of collaboration between government agencies and private industries (PPP) for the development of CDW recycling plants and formal markets for recycled materials • Enforcement of using certain percentage of recycled materials in public construction projects to create a market demand	DoE, PWD, City management authorities (i.e., RAJUK), LGED, public and private construction sector
*Technological* • Limited availability of advanced recycling technologies for processing solid waste as well as CDW efficiently • Lack of technical expertise and skilled workforce in handling and processing recycled waste materials • Poor quality control and standardization of recycled aggregates, affecting their acceptance in construction • Need for research and development to enhance the durability and usability of recycled materials in large-scale infrastructure projects	*Improving data collection and waste tracking* • Development of national database where construction bodies update on CDW generation, disposal, and recycling activities in the projects • Assessment of waste composition, volume, and recycling potential through mandatory periodic audits for urban development projects • Inclusion of waste management as a major component of city planning to ensure efficient waste processing and recycling facilities in high-growth areas • Arrangement of technical training programs for construction professionals on sustainable building materials and waste management strategies • Promotion of education and outreach programs to encourage sustainable construction practices among developers, contractors, landowners and public	MoPSID of GoB, public and private construction sector

The primary aim of the United Nations Sustainable Development Goals (SDGs) is to create a balance between environmental protection and development at all phases. As the construction industry helps a nation's economic growth, it has a responsibility to prevent environmental deterioration, and many of the SDGs are closely connected to this sector. Specifically, Target 11.6 focuses on reducing the negative effects of cities on the environment per person by 2030, particularly by focusing on air quality and managing municipal and other waste. While sustainable construction practices are prevalent in industrialized nations, the proportion of eco-friendly projects in underdeveloped nations is far smaller. Table 10 presents a few of the notable SDGs concerning the CDW sectors regarding the environment and global climate change issues.

**Table 10:** Relevant SDGs connected with the sustainability of the construction industry

Goals	Topic	Way to achieve
SDG 6	Clean water and Sanitation	• The construction industries need to take the appropriate precautions against the potential contamination of freshwater bodies around their premises. Additionally, they need to ensure that waste is not disposed of in the neighboring water body
SDG 9	Industry, Innovation, and Infrastructure	• Construction companies need to assess the social and environmental risks posed by CDW and assess how they can minimize them. Encouraging innovation in waste reduction techniques and technologies can result in construction processes, which are both eco-friendly and productive
SDG 11	Sustainable Cities and Communities	• Minimizing construction waste reduces the pressure on local waste management systems in urban areas. Implementing recycling and waste reduction measures in construction projects fosters sustainable urban development • Strategies to reduce construction waste can enhance resource efficiency and promote the use of recycled materials, contributing to greener cities
SDG 12	Responsible consumption and production	• Promoting waste reduction in construction aligns with the goal of sustainable consumption and production. Adopting circular economy principles in construction can reduce the extraction of raw materials and minimize landfill waste
SDG 13	Climate action	• By using energy-efficient procedures, cutting waste production, and integrating renewable energy sources, construction supply chains can lower their GHG emissions

This study offers a data-driven, field assessment of waste generation in a rapidly growing urban region of Bangladesh, which could function as a model for other developing countries encountering similar problems with CDW management. The study identifies key barriers in Table 9, providing a working framework for policymakers in South Asia and other developing regions to formulate context-specific CDW recycling policies. The findings may also enhance international initiatives such as SDGs 9, 11 and 12 by advancing resource efficiency and CE principles. This study's insights are expected to help global organizations such as the UN-Habitat, the World Bank, and the International Solid Waste Association (ISWA) in developing waste management strategies specifically designed for emerging and developing economies.

## Conclusion

The present study was carried out to investigate WGRs of CDW in the Greater Dhaka area of Bangladesh from 21 construction and 12 demolition sites, along with the recycling potential of the studied waste components for the eight major districts of this region. Efforts to protect the environment from land disposal of CDW were investigated as well. Research findings show that the WGR for demolition and construction sites is found to be 463.67 kg/m^2^ and 90.31 kg/m^2^, respectively. The major components in CDW are mainly composed of concrete, brick, and mortar in both construction and demolition sites. From the physio-chemical analysis of the selected samples, the heavy metal concentrations of Pb, Cd, and Cr do not exceed standards set by the US EPA which suggests that the waste components are safe regarding future handling for recycling and reuse. Additionally, the findings indicate that the WGR and the recycling potential of CDW are statistically significant (r = 0.97). The economic benefit analysis of recycling CDW showed that the most profit can be generated from metal > brick > concrete waste components from both construction and demolition sites. In addition, the study offers a discussion on efficient CDW management, recycling and recovery of waste components as well as business development through the 3R and CE approaches. This study uniquely contributes to the existing knowledge on CDW management by concentrating on the Greater Dhaka area, utilizing a longitudinal analysis, employing a multifaceted methodology, conducting a comprehensive economic benefit analysis, and highlighting business development and policy implications. The findings are expected to assist legislators, urban planners, engineers and the overall construction sector in incorporating recycling facilities into municipal development strategies. By addressing the identified challenges (economic, technological, and regulatory), participating agencies can advocate for regional collaborations through a multi-stakeholder approach, supporting the construction industry's transition toward sustainable development.

## Data Availability

Data will be made available upon reasonable request to the corresponding author.

## Appendix 2

Table 11

**Table 11:** Projected increase in land area from years 2018–2030 in the studied eight districts of Greater Dhaka area

	Projected land area in every year (2018–2030) in m^2^												
Districts	2018	2019	2020	2021	2022	2023	2024	2025	2026	2027	2028	2029	2030
Dhaka city	170882483.95	194867022.5	222217956.9	253407784	288975319.4	329535004.2	375787521.2	428531899	488679315	557268836.8	635485372.5	724680139.9	826394009.8
Gazipur	132812610.43	201498623.4	305706627.5	463807348	703672202.3	1067586727	1619705050	2457359559	3728219532	5656323604	8581575317	13019664376	19752977070
Savar	121892661.88	253546433.8	527396753	1.097E + 09	2281903855	4746541553	9873184038	20537008253	42718610973	88858109,269	1.84832E + 11	3.84465E + 11	7.99718E + 11
Narayanganj	80423957.95	121556343	183725657.2	277691121	419714696.7	634375439.2	958823222.1	1449208015	2190397378	3310663910	5003884515	7563093363	11431195313
Keraniganj	38100332.12	58842128.41	90875745.24	140348442	216754044.2	334754806.3	516995107.4	798446911.2	1233120896	1904431118	2941202192	4542390773	7015265386
Bandar	30940947.76	49783788.27	80101798.87	128883285	207372386.6	333660851.9	536858189.8	863801414.8	1389850986	2236261401	3598130379	5789368908	9315057772
Rupganj	33,372,356.78	63064224.07	119173374	225203644	425570575.5	804206856.3	1519721299	2871839263	5426956087	10255397212	19379772066	36622234865	69205565572
Sonargaon	18388708.95	33,984,383.6	62806928.54	116074204	214518065.6	396453293.7	732689872.4	1354092544	2502513938	4624924669	8547376246	15796503927	29193699814

Calculated after Wang and Sarker (2020)

## Appendix 3

Table 12

**Table 12:** Estimated total construction and demolition floor areas in the studied eight districts of Greater Dhaka from years 2018–30

	Construction floor area (m^2^)												
Districts	2018	2019	2020	2021	2022	2023	2024	2025	2026	2027	2028	2029	2030
Dhaka city	2734415	2953168	3189421	3444575	3720141	4017753	4339173	4686307	5061211	5466108	5903397	6375668	6885722
Gazipur	2850001	3289591	3956522	4968367	6503502	8832555	12366112	17727101	25860605	38200471	56922082	85325852	1.28E + 08
Savar	2780113	3622697	5375339	9020974	16604185	32377866	65188378	1.33E + 08	2.75E + 08	5.71E + 08	1.18E + 09	2.46E + 09	5.12E + 09
Narayanganj	2514713	2777961	3175844	3777223	4686174	6060003	8136469	11274931	16018543	23188249	34024861	50403798	75159650
Keraniganj	2243842	2376590	2581605	2898230	3387226	4142431	5308769	7110060	9891974	14188359	20823,94	31071301	46897698
Bandar	2198022	2318616	2512652	2824853	3327183	4135429	5435892	7528329	10895046	16312073	25028034	39051961	61616370
Rupganj	2213583	2403611	2762710	3441303	4723652	7146924	11726216	20379771	36732519	67634542	1.26E + 08	2.36E + 08	4.45E + 08
Sonargaon	2117688	2217500	2401964	2742875	3372916	4537301	6689215	10666192	18016089	31599518	56703208	1.03E + 08	1.89E + 08
	Demolition floor area (m^2^)												
Dhaka city	816772.8	882114.6	952683.8	1028898	1111210	1200107	1296116	1399805	1511789	1632733	1763351	1904419	2056773
Gazipur	783460	913963.4	1111959	1412350	1868093	2559531	3608556	5200099	7614733	11278131	16836109	25268478	38061772
Savar	762712.1	1012854	1533170	2615468	4866733	9549545	19290166	39551432	81696477	1.69E + 08	3.52E + 08	7.31E + 08	1.52E + 09
Narayanganj	683921.5	762073.1	880194.7	1058729	1328574	1736429	2352880	3284611	4692871	6821377	10038497	14900993	22250387
Keraniganj	603506.6	642916	703779.9	797778	942948.7	1167,150	1513407	2048165	2874046	4149535	6119400	9161658	13860120
Bandar	589903.8	625705.2	683309.4	775994.2	925123.5	1165072	1551147	2172339	3171833	4780013	7367564	11530917	18229726
Rupganj	594523.5	650938	757545.4	959002.9	1339700	2059109	3418586	5987611	10842333	20016371	37352683	70113362	1.32E + 08
Sonargaon	566054.5	595686.3	650449.2	751657	938700.3	1284377	1923227	3103892	5285892	9318473	16771131	30544473	55999146

## Appendix 4

Table 13

**Table 13:** Identified recycling potential and values of the CDW items based on the field survey

Waste items	Project type	Avg. WGRi (kg/m^2^)	WC (%)	Recycling rate	Recycling potential (kg/m^2^)	Recycling value (BDT/tonne) ^a^
Cement	DP	50.67	10.93	0.70	3.88	100.00
	CP	10.00	11.08	0.70	0.78	100.00
Brick	DP	131.14	28.28	0.80	29.67	1820.00
	CP	26.00	28.79	0.80	5.99	1820.00
Metal	DP	32.42	6.99	1.00	2.27	50000.00
	CP	5.42	6.01	1.00	0.33	50000.00
Concrete	DP	188.10	40.57	0.70	53.41	188.00
	CP	37.13	41.12	0.70	10.69	188.00
Mortar	DP	61.35	13.23	0.70	5.68	188.00
	CP	11.75	13.01	0.70	1.07	188.00

Source: ^a^Haque et al. (2024)

## Appendix 5

Figures 7 and 8

## Abbreviations

BIM: Building Information Modeling
CDW: Construction and Demolition Waste
CE: Circular Economy
CP: Construction project
DoE: Department of Environment
DP: Demolition project
EU: European Union
GHG: Greenhouse Gas
GIS: Geographic Information System
GoB: The Government of Bangladesh
ISWA: International Solid Waste Association
LGED: Local Government Engineering Department
MoPSID: Ministry of Planning, Statistics and Informatics Division
MSW: Municipal Solid Waste
PPP: Public-Private Partnership
PVC: Polyvinyl Chloride
PWD: Public Works Department
RAJUK: Rajdhani Unnayan Kartripakkha
RCC: Reinforced Cement Concrete
SDGs: Sustainable Development Goals
US EPA: The United States Environmental Protection Agency
WGR: Waste Generation Rates
